# Pharmacovigilance and Appropriate Drug Use

**DOI:** 10.3390/healthcare12060669

**Published:** 2024-03-16

**Authors:** Lucia Gozzo

**Affiliations:** Clinical Pharmacology Unit, Regional Pharmacovigilance Centre, Azienda Ospedaliero Universitaria Policlinico “G. Rodolico—S. Marco”, 95123 Catania, Italy; luciagozzo86@icloud.com

This Special Issue collects updated evidence about pharmacovigilance and regulatory actions which can be translated into the change and control of prescribing behaviour.

Pharmacovigilance is a key public health process which helps in monitoring the safety of medicines and reducing the risks related to drug use in the post-marketing phase (phase IV) [1,2,3,4,5,6,7,8].

The emergence of safety signals in this phase may result in regulatory action, translating into modification of the terms of the marketing authorization or the way the drug can be used to protect public health [9,10,11,12,13,14,15,16,17,18,19,20,21,22,23,24,25].

Nevertheless, the safety of a medicine is related not only to its pharmacological properties, but also to the product’s quality and to how it is used in clinical practice. According to the World Health Organization (WHO), inappropriate drug use has been reported for more than 50% of all medicines in the market and results in various health risks, including the onset of adverse events [26].

The appropriate prescribing, dispensing, and use of drugs include several steps performed both by healthcare professionals and by patients.

Therefore, appropriate drug use helps to avoid medication errors and increase drug safety.

Regulatory agencies can use several tools to control and manage each step of the process in order to guarantee the appropriateness of medicine use.

Sardeli et al. (Contribution 1) investigated the role of community pharmacies to detect errors in prescribing, drug–drug interactions, and patients’ compliance. A significant number of patients (16%) did not comply with prescribed pharmacotherapy, and 32% used drugs for self-medication. In total, 9% of prescriptions included drugs with potentially severe drug–drug interactions and 59% with moderate interactions. Community pharmacies are crucial in the detection and evaluation of prescribing errors and pharmacotherapy problems.

The review by Gozzo et al. (Contribution 2) underlines the criticism of drug substitution in the particular case of generics of drugs with a narrow therapeutic index (NTI). Moreover, generics cannot automatically be considered bioequivalent each other due to the biocreep phenomenon. These issues should be taken into account by healthcare professionals in order to guarantee patients’ safety.

In these regards, the study by Alsufyani et al. (Contribution 3) aims to understand the role of hospital pharmacists for generic substitution, a pharmacist-initiated act usually justified by medication unavailability, side effects, patient characteristics, outcomes, and economic reasons. This practice is recognized as a valuable support to improve patients’ outcomes.

Moreover, pharmacovigilance awareness is a key factor in improving the culture of adverse drug reaction (ADR) reporting.

Valinciute et al. (Contribution 4) aim to understand the consumer knowledge regarding ADRs and factors impacting their engagement in reporting with a questionnaire-guided cross-sectional survey among 404 patients. This study demonstrates a generally poor understanding of Lithuanian consumers of pharmacovigilance, but a favourable attitude toward ADR reporting. The results can help to develop educational interventions to address current limitations in the culture of pharmacovigilance.

Real-world evidence (RWE) represents an important data source which can complement evidence from clinical trials and support post-approval assessments by competent authorities, particularly in pharmacovigilance.

Alhlayl et al. (Contribution 5) analysed data from Swansea University’s United Kingdom (UK) Multiple Sclerosis (MS) Register, a platform containing information of more than 17,600 people with MS. The aim of the study was to elucidate the trends in the patterns of medicines used by people with MS.

The frequency rates of disease-modifying drugs (DMDs) used in this study ranged from 1% to 4%. Moreover, to manage associated symptoms such as pain, spasticity, fatigue, bladder dysfunction, and depression, patients used concomitant medications including muscle relaxants, anticonvulsants, antidepressants, and antianxiety medicines.

Han et al. (Contribution 6) assessed the patterns of pharmacological treatment for outpatients with postherpetic neuralgia (PHN), from the database of the Hospital Prescription Analysis Program of China.

Gabapentin and pregabalin were the most commonly prescribed drugs, in accordance with current guidelines, followed by opioids. The frequent use of oxycodone raised concerns about appropriateness, also from the economic point of view.

The study by Mattioli et al. (Contribution 7) showed the adverse events (AEs) related to co-treatment with ACE inhibitors (ACE-I), and/or angiotensin receptor blockers (ARBs), with diuretics and non-steroidal anti-inflammatory drugs (NSAIDs), leading to emergency department (ED) visits and/or hospitalisations in Italy, using the MEREAFaPS database. Polypharmacy can lead to serious AEs, poor adherence, and drug–drug interactions. In the reference period, 80 patients visited the ED for AEs, and 47 were hospitalised (58.75%), but no significant differences in the risk of hospitalisation were found according to demographic, clinical, or pharmacological features. In total, 261 suspected drugs were identified, and drug–drug interactions were associated with kidney injury.

Adherence is another crucial parameter for treatment outcomes in several medication settings.

Inoue et al. (Contribution 8) conducted an anonymous self-administered web-based survey to study real-world treatment adherence in 1030 Japanese people living with HIV (PLHIV), critical for antiretroviral therapy (ART) efficacy. Among 821 PLHIV who responded to the survey, 291 (35%) were included in the low adherence group. Risk factors for low adherence were young age, moderate to severe depression, and drug dependence. Adherence was also influenced by a shared decision-making process; therefore, better support from care providers should be considered critical for improving adherence.

The study by Mucherino et al. (Contribution 9) aims to adapt the original Ascertaining Barriers for Compliance (ABC) Taxonomy on medication adherence for the Italian language. The Taxonomy is useful in research, academic, and professional fields in order to harmonize adherence terminology.

Finally, Gozzo et al. (Contribution 10) report the updated safety data of gilteritinib in patients with acute myeloid leukemia (AML) [27]. In particular, the study is focused on the gastrointestinal (GI) toxicity of the drug, indicated as very common in the summary of product characteristics, even if the association has not been confirmed. Indeed, serious GI AEs represent an important potential risk to be monitored in postmarketing surveillance in patients treated with gilteritinib.

## List of Contributions

1. Sardeli, C.; Athanasiadis, T.; Stamoula, E.; Kouvelas, D. Pharmacologic Stewardship in a Rural Community Pharmacy. *Healthcare* **2023**, *11*, 2619. https://doi.org/10.3390/healthcare11192619.
2. Gozzo, L.; Caraci, F.; Drago, F. Bioequivalence, Drugs with Narrow Therapeutic Index and the Phenomenon of Biocreep: A Critical Analysis of the System for Generic Substitution. *Healthcare* **2022**, *10*, 1392. https://doi.org/10.3390/healthcare10081392.
3. Alsufyani, M.H.; Alghoribi, M.H.; Bin Salman, T.O.; Alrabie, A.F.; Alotaibi, I.S.; Kharbosh, A.M.; Alsheikh, M.Y.; Alshahrani, A.M.; Fathelrahman, A.I. Generic Substitutions and Therapeutic Interchanges in Hospital Pharmacies: A Qualitative Study from Western Saudi Arabia. *Healthcare* **2023**, *11*, 1893. https://doi.org/10.3390/healthcare11131893.
4. Valinciute, A.; Gerbutaviciene, R.J.; Paukstaitiene, R.; Kubiliene, L. Pharmacovigilance and Adverse Drug Reaction Reporting among the General Public in Lithuania: A Cross-Sectional Study. *Healthcare* **2023**, *11*, 1133. https://doi.org/10.3390/healthcare11081133.
5. Alhlayl, A.S.; Alzghaibi, H.A.; Jamal, Q.M.S. Determining Current Medications Usage within a Cohort of Patients in the UK—A Descriptive Retrospective Study. *Healthcare* **2022**, *10*, 2421. https://doi.org/10.3390/healthcare10122421.
6. Han, G.; Han, Y.; Yu, L.; Zhao, Y.; Yu, Z. Patterns and Trends in Pharmacological Treatment for Outpatients with Postherpetic Neuralgia in Six Major Areas of China, 2015–2019. *Healthcare* **2023**, *11*, 764. https://doi.org/10.3390/healthcare11050764.
7. Mattioli, I.; Bettiol, A.; Crescioli, G.; Bonaiuti, R.; Mannaioni, G.; Vannacci, A.; Lombardi, N. on behalf of the MEREAFaPS Study Group. Hospitalisations Related to the Combination of ACE Inhibitors and/or Angiotensin Receptor Blockers with Diuretics and NSAIDs: A Post Hoc Analysis on the Risks Associated with Triple Whammy. *Healthcare* **2023**, *11*, 238. https://doi.org/10.3390/healthcare11020238.
8. Inoue, Y.; Oka, S.; Yokoyama, S.; Hasegawa, K.; Mahlich, J.; Schaede, U.; Habuka, N.; Murata, Y. Medication Adherence of People Living with HIV in Japan—A Cross-Sectional Study. *Healthcare* **2023**, *11*, 451. https://doi.org/10.3390/healthcare11040451.
9. Mucherino, S.; Maffoni, M.; Cena, C.; Armando, L.G.; Guastavigna, M.; Orlando, V.; Orofino, G.; Traina, S.; Giardini, A.; Menditto, E.; et al. Italian Translation and Validation of the Original ABC Taxonomy for Medication Adherence. *Healthcare* **2023**, *11*, 846. https://doi.org/10.3390/healthcare11060846.
10. Gozzo, L.; Nardo, A.; Brancati, S.; Judica, A.; Duminuco, A.; Maugeri, C.; Parisi, M.; Longo, L.; Vitale, D.C.; Ruscica, R.; et al. Severe Gastrointestinal Toxicity Following the Use of Gilteritinib: A Case Series and Analysis of Postmarketing Surveillance Data. *Healthcare* **2023**, *11*, 1479. https://doi.org/10.3390/healthcare11101479.
